# Molecular tests for human papillomavirus (HPV), *Chlamydia trachomatis *and *Neisseria gonorrhoeae *in liquid-based cytology specimen

**DOI:** 10.1186/1472-6874-9-8

**Published:** 2009-04-09

**Authors:** Sin Hang Lee, Veronica S Vigliotti, Suri Pappu

**Affiliations:** 1 Department of Pathology, Milford Hospital, Milford, Connecticut, USA

## Abstract

**Background:**

Laboratory detection of Human papillomavirus (HPV), *Chlamydia trachomatis *and *Neisseria gonorrhoeae *in liquid-based cervicovaginal cytology specimens is now based on identification of the DNA sequences unique to these infectious agents. However, current commercial test kits rely on nucleotide probe hybridization to determine DNA sequences, which may lead to diagnostic errors due to cross-reactivity. The aim of this study was to find a practical approach to perform automated Sanger DNA sequencing in clinical laboratories for validation of the DNA tests for these three infectious agents.

**Methods:**

A crude proteinase K digestate of 5% of the cells collected in a liquid-based cervicovaginal cytology specimen was used for the detection of DNA molecules specific for HPV, *C trachomatis *and *N gonorrhoeae*, and for preparation of materials suitable for direct automated DNA sequencing. Several sets of commercially available polymerase chain reaction (PCR) primers were used to prepare nested PCR amplicons for direct DNA sequencing.

**Results:**

Some variants of HPV-16 and HPV-31 were found to share an at least 34-base long sequence homology downstream of the GP5+ binding site, and all HPV-6 and HPV-11 variants shared an upstream 34-base sequence including part of the GP5+ primer. Accurate HPV genotyping frequently required more than 34-bases for sequence alignments to distinguish some of the HPV genotype variants with closely related sequences in this L1 gene hypervariable region. Using the automated Sanger DNA sequencing method for parallel comparative studies on split samples and to retest the residues of samples previously tested positive for *C trachomatis *and/or for *N gonorrhoeae*, we also found false-negative and false-positive results as reported by two commercial nucleic acid test kits.

**Conclusion:**

Identification of a signature DNA sequence by the automated Sanger method is useful for validation of HPV genotyping and for molecular testing of *C trachomatis *and *N gonorrhoeae *in liquid-based cervicovaginal Papanicolaou (Pap) cytology specimens for clinical laboratories with experience in molecular biology to increase the specificity of these DNA-based tests. However, even a highly specific test for high-risk HPV genotyping may have unacceptably low positive predictive values for precancer lesion in populations with a low cervical cancer prevalence rate.

## Background

Human papillomavirus (HPV), *Chlamydia trachomatis *and *Neisseria gonorrhoeae *are the causative agents for the three most common sexually transmitted infections in women. Newly introduced laboratory diagnostic procedures for these infectious agents are mostly nucleic acid-based, relying on detection and identification of a DNA sequence specific for the infectious agent with or without DNA replication (amplification) by polymerase chain reaction (PCR). The tests incorporating PCR amplification in the procedure, often referred to as nucleic acid amplification (NAA) tests, are extremely sensitive, capable of detecting a single copy of target DNA. The result of a DNA-based test may be validated by identifying a signature sequence of the target DNA and used for accurate genotyping if HPV DNA is the target [1-8].

The commercial NAA tests for *C trachomatis *and *N gonorrhoeae*, all relying on probe hybridization for DNA sequence determination, are claimed to have a sensitivity of 85% and a specificity of 97–99%, depending on the evaluating method chosen for comparison [9]. These tests tend to generate unacceptably low positive predictive value (*PPV*) [10] in low prevalence populations. As a result, the Centers for Disease Control and Prevention (CDC) has issued recommendations that an additional test with different method or with the same method be performed to confirm a positive NAA test for *C trachomatis *and *N gonorrhoeae *to reduce the possibility of false-positive results that may have adverse medical, social and psychological impacts on the patient, but also cautions that such a supplementary test result might itself be falsely negative [9]. Clinical laboratories often find these tests are associated with 5.3% of initially *C trachomatis*-positive results and 10.7% of initially *N gonorrhoeae*-positive results that cannot be confirmed by repeated testing of the original sample [11].

In the past few years the cost of performing automated DNA sequencing has decreased considerably. This article gives a brief summary on the experience of applying this research tool in HPV genotyping and in the identification of *C trachomatis *and *N gonorrhoeae *DNA in liquid-based Pap cytology specimens to serve the physicians who want unequivocal evidence to validate each test result.

## Methods

### Clinical Materials

The clinical samples used for the HPV study were 2,020 alcohol-preserved liquid-based cervicovaginal Pap cytology specimens (ThinPrep or Surepath) routinely submitted by 2 private gynecologists who provided general obstetric and gynecologic care to local residents in Milford, Connecticut. Milford has a rural and suburban population of 50,000+, in which 93.6% of the residents are non-Hispanic whites according to the latest available census [12]. The incidence of cervical cancer in the 2,020 patients selected for this study was considered to be representative of or below that in the state of Connecticut in which the cervical cancer rate is 6.8 per 100,000 women [13]. Publication of the laboratory data with blinded patient identities was approved by the Milford Hospital IRB.

**Figure 1:**
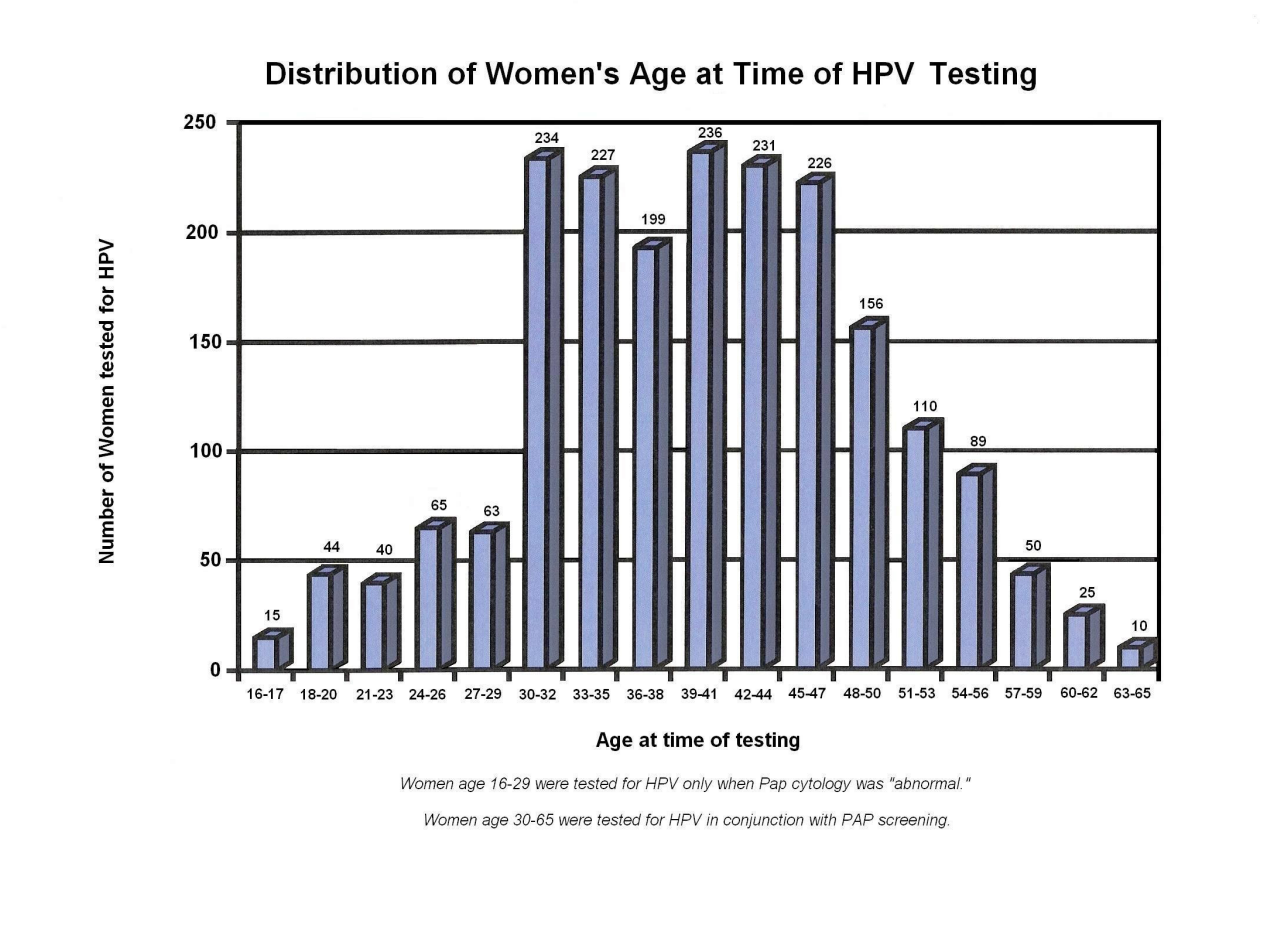
**Age distribution of 2,020 private patients tested for HPV in Milford, CT**.

The cervicovaginal specimens were collected from women below age 30 who had a Pap cytology finding of atypical squamous cells of undetermined significance (ASCUS) or more severe, and from women 30 years and older in conjunction with Pap cytology screening when the clinical specimens were submitted for HPV testing with or without concomitant request for *C trachomatis *or *N gonorrhoeae *testing (Figure 1). The Pap cytology slides were screened by a commercial cytology laboratory and all abnormal slides reviewed by the 2 author pathologists for final classification.

The cases classified as "ASCUS favoring a reactive process" under the Bethesda system [14] were grouped with the normal cases under less than ASCUS (<ASCUS) for the purpose of analysis in this report. Samples submitted in the liquid-based Pap cytology vial for *C trachomatis *and/or *N gonorrhoeae *testing were generally from "asymptomatic" women 15 to 65 years old regardless of Pap cytology findings.

A total of 510 liquid-based Pap cytology specimens were tested for *C trachomatis *DNA and a total of 507 tested for *N gonorrhoeae *DNA.

### Primary and Nested PCR

The cellular debris derived from 5% of a liquid-based Pap cytology collection was pelleted for primary and nested PCR amplification of HPV, *C trachomatis *or *N gonorrhoeae *DNA without DNA extraction or purification, as previously described [15-17].

The residues of 26 endocervical samples which had been tested positive by the BD Probe Tec™ ET *Chlamydia trachomatis *and *Neisseria gonorrhoeae *Amplified DNA Assays (Becton, Dickinson, Sparks, MD) were retested by DNA sequencing for comparison [16].

All positive nested PCR products were validated by on-line Basic Local Alignment Search Tool (BLAST) algorithms for final validation of the signature sequence.

The numbers of cases analyzed did not include 38 cases which were considered inadequate for failed PCR amplification of a human β-globin genomic DNA in the samples.

## Results

**Figure 2:**
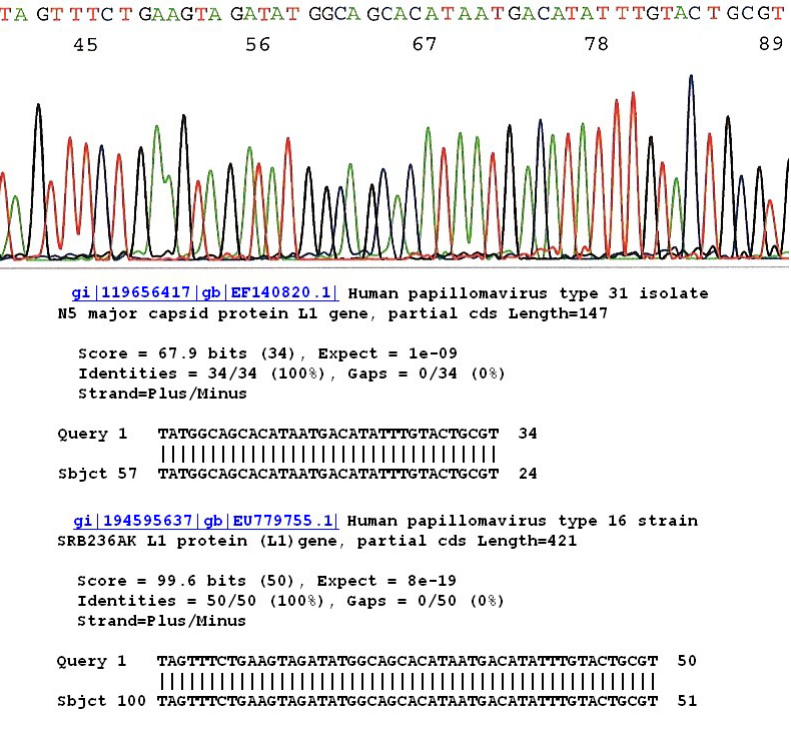
**Genotyping distinction between HPV-16 and HPV-31 may need a 50-base sequence**. HPV DNA isolated from a clinical sample with its partial L1 gene DNA sequence of 50 bases is validated as HPV-16 strain SRB236AK L1 protein (L1) gene by the GenBank database. However, HPV-31 isolate N5 and some HPV-33 major capsid protein L1 genes share an identical 34-base sequence on the upstream (right) segment in this region with this variant of HPV-16. Genotyping error might have occurred if only the 34 bases on the right were selected for sequence alignment algorithm.

**Figure 3:**
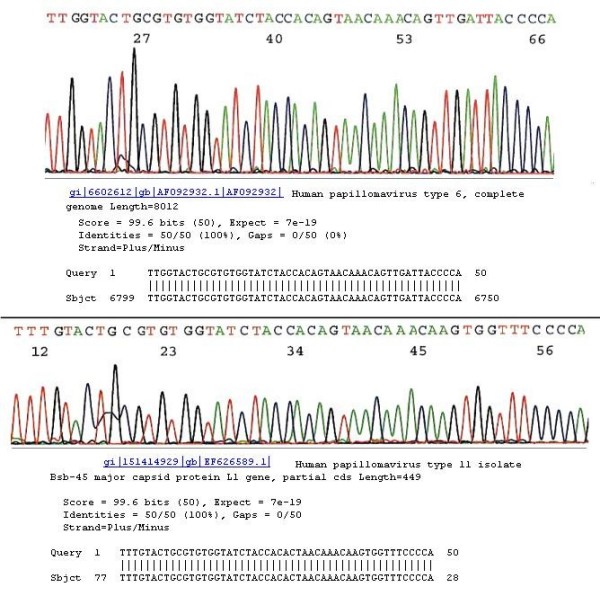
**All HPV-6 and HPV-11 isolates share common L1 DNA sequences**. Fig. 3 upper HPV-6 DNA isolate from a clinical sample with its partial L1 gene DNA sequence of 50 bases is validated as HPV-6 by the GenBak database. Fig. 3 lower HPV-11 DNA isolate from a clinical sample with its partial L1 gene DNA sequence of 50 bases is validated as HPV-11 isolate Bsb-45 major capsid protein by the GenBak database. From the left No. 4 base onward (upstream), all known HPV-6 (upper) and HPV-11 (lower) isolates share a 34-base sequence. In this "hypervariable region" which is generally used for HPV L1 genotyping, differentiation between HPV-6 and HPV-11 depends on identification of a four-base difference upstream of these 34 common bases, but before another sequence homogeny starting with CCCCA.

For the samples in which there was a single HPV genotype in the specimen, genotyping was readily determined with direct automated DNA sequencing by on-line BLAST sequence alignment algorithms. An exclusive 100% "identities" match between the "query" sequence and the "subject" sequence was required for accurate genotyping. A 34-base sequence downstream of the GP5+ primer site excised from the electropherogram of the sample, which is fully matched with a standard HPV signature sequence stored in the GenBank validated the HPV genotype, except for some variants of HPV-16, HPV-31 and HPV-33, for which BLAST algorithms of a 46–50 base sequence in this region were needed for unequivocal genotyping (Fig. 2). When the GP6/MY11 or the HiFi nested PCR primer pair (catalog #3002, HiFi DNA Tech, LLC, Trumbull, CT) was used to replace the GP5+/GP6+ primers to prepare a 190–200 bp nested PCR amplicon for DNA sequencing, an upstream 30- to 34-base long segment including 11 bases of the GP5+ primer site, was found to be sufficient to differentiate these variants. However, all variants of HPV-6 and HPV-11 share one DNA sequence homology in this region for 34 bases. Differentiation between these latter two HPV genotypes depended on finding the four isolated distinguishing bases further upstream of this sequence homology (Fig. 3).

Among 2020 specimens, 200 (9.9%) were positive for HPV DNA, all confirmed by DNA sequencing. A total of 30 genotypes were found, including 12 of the 13 "high-risk" genotypes targeted by the Digene HC2 assay (Digene Corporation, Gaithersburg, MD), except HPV-68 which was detected in this County, but not in this series. Of the 200 HPV-positive specimens, 183 (91.5%) were infected by a single HPV genotype, all assigned a specific genotype according to the GenBank sequence database BLAST algorithms, and 17 positive samples (8.5%) contained more than one HPV genotype (Additional file 1, Table 1). Since the patients under age 30 were pre-selected by an abnormal Pap cytology, the HPV positive rate in this group was about 5 times that observed among the patients age 30 or older [17].

Of the 200 HPV-positive specimens, 119 (59.5%) were found to contain at least one of the 13 genotypes of HPV targeted by the Digene HC2 "high-risk" HPV test kit, including 3 cases of mixed infections by an HPV-16, HPV-18 or both, confirmed by specific primer sequencing [18]. Of the HPV-positive specimens, 67 (33.5%) contained one of the HPV types other than the 13 targeted by the HC2 kit.

Four (4) cases of HSIL (high-grade squamous epithelial lesion) were found in this series, constituting 0.2% of the specimens submitted for HPV testing, namely in 2% of the specimens with a positive result for HPV. Two of these HSILs were associated with a single HPV-16, one with an HPV-39, and one with an HPV-69 infection. A LSIL (low-grade squamous epithelial lesion) was found in 29 of the 200 specimens with a positive result for HPV (14.5%). The 123 cases classified as "<ASCUS" included the specimens with a normal Pap cytology from women age 30 or older, and those specimens from younger women whose Pap cytology was classified as "ASCUS favoring a reactive process".

**Figure 4:**
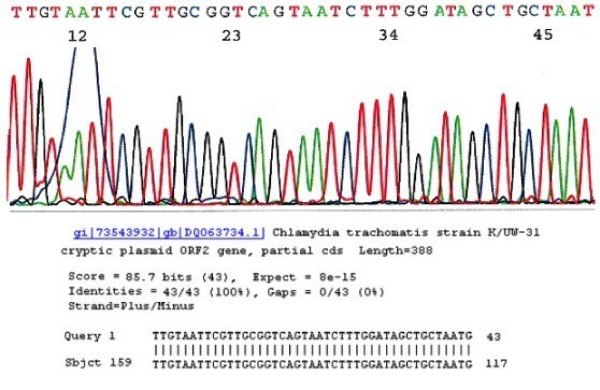
**Species-specific signature sequence of *Chlamydia trachomatis *cryptid plasmid DNA**. The signature sequence for *Chlamydia trachomatis *is almost constant between various clinical isolates. Any 40 bases selected from the electropherogram of this DNA sequence of the positive nested PCR product gives a "100% identities" match for a cryptic plasmid gene.

A 40-base signature sequence of the cryptic plasmid DNA specific for the *C trachomatis *species was used as a signature sequence (Fig. 4). A visualized nested PCR product finally proved to be *C trachomatis *cryptic plasmid DNA by sequencing in the absence of a concomitant primary PCR band represented detection of a "low-positive" amount of species-specific DNA equivalent to that extracted from 1 to 2 × 10^6 ^elementary bodies in one sample collection. The positive cases with a concomitant visible primary PCR band were classified as "high-positive" [16].

**Figure 5:**
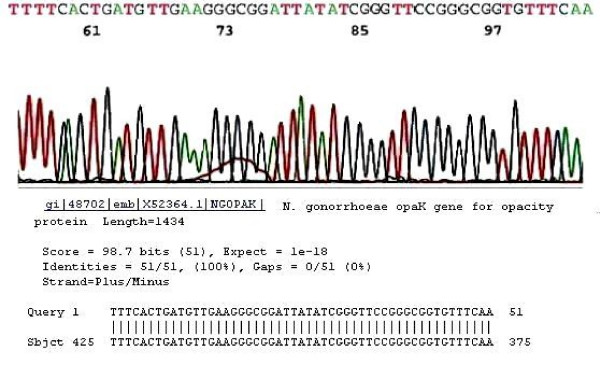
**Species-specific sequences of *Neisseria gonorrhoeae opa *genes**. The electropherogram of gonococcal *opa *genes in this signature sequence region always illustrates a mixture of sequences generated by multiple *opa *gene templates. This particular tracing shows a clear base calling of 50 bases by the computer. However, in many cases only 40 bases on the right are selected for analysis because overlapping peaks tend to appear toward the left of the tracing due to co-migration of nucleotides having one molecular weight, but carrying different dye-labeled terminators due to the fact that gonococcal *opa *gene nested PCR products are not homogenous and may range from 127 to 130 base pairs in size.

A 40-base DNA sequence on the electropherogram was found to be sufficient for validation of the signature sequence of gonococcal *opa *genes (Fig. 5). A "High-positive" result, indicated by a concomitant visible primary PCR product, represented detection of a DNA content extracted from at least 2 × 10^5 ^cells of *N gonorrhoeae *in one sample collection. Positive cases detected by nested PCR only were classified as "low positive" [16].

Of the clinical specimens collected from 146 patients, 2 in paired samples for parallel assays by PCR/DNA sequencing and by the GEN-PROBE^® ^PACE^® ^2C test were found to be "high-positive" for *C trachomatis *cryptic plasmid DNA by nested PCR/DNA sequencing and also positive by the GEN-PROBE^® ^PACE^® ^2C kit assay for *C trachomatis*. Another four paired specimens (4/146) were found to be "low-positive" for *C trachomatis *cryptic plasmid DNA by nested PCR/DNA sequencing, but negative by GEN-PROBE^® ^assays. All remaining 140 samples were negative for *C trachomatis *by both methods.

All 146 paired samples submitted for Gen-Probe assays were found to be negative for *N gonorrhoeae*. But 5 of the146 liquid-based Pap cytology specimens (5/146) submitted for nested PCR/DNA sequencing were found to be "low-positive" for gonococcal *opa *gene DNA by nested PCR/DNA sequencing.

The residues of 26 endocervical samples previously tested positive by the BD Probe Tec™ ET *Chlamydia trachomatis *and *Neisseria gonorrhoeae *Amplified DNA Assays (Becton, Dickinson and Co., Sparks, MD) were retested for comparison, following a protocol previously published [16]. The results showed that 8 of the 14 cases reported to be positive for *C trachomatis *by the BD Probe Tec assays were found to be "high-positive", 5 of the 14 to be "low-positive", and 1 of the 14 to be negative for *C trachomatis *cryptic plasmid DNA. Of the 12 cases reported to be positive for *N gonorrhoeae *by the BD Probe Tec assays, 8 were found to be "high-positive", and 4 to be "low-positive" for gonococcal gene DNA. In addition, nested PCR/DNA sequencing showed that one of the *N gonorrhoeae *"high-positive" cases also contained a "high-positive" *C trachomatis *cryptic plasmid DNA which was in a mixed infection and missed by the BD Probe Tec test. Thus, the nested PCR/DNA sequencing retesting of the residues of 26 specimens reported to be positive by a commercial NAA test kit found 1 false positive and 1 false negative result.

Using the nested PCR/DNA sequencing assays on samples collected in liquid-based Pap cytology vials, we found 2 cases of "high-positive" (0.39%) and 5 cases of "low-positive" (0.98%) *C trachomatis *infection in 510 consecutively received routine specimens collected from 2 private medical offices in Milford. The two "high-positive" specimens were collected from a patient presenting with a recent onset of lower abdominal pain and from a patient whose male sexual partner was recently diagnosed with acute chlamydial urethritis. During the same period, we found 10 "low-positive" (1.97%) and no "high-positive" (0%) *N gonorrhoeae *cases in 507 consecutively received liquid-based Pap cytology specimens.

## Discussion

Unlike the traditional methods based on bacterial culture, the highly sensitive commercial NAA tests for detecting *C trachomatis *and *N gonorrhoeae *lack a 100% analytical specificity [9]. No commercial test kits available in the USA for specific genotyping of the 13 generally recognized high-risk HPV genotypes.

Several probe-based test kits for HPV genotyping have been evaluated [19,20] for information on accurate HPV genotyping may play an important role in following persistent HPV infections [21-24]. However, when the GP5+/GP6+ PCR products with a hypervariable DNA sequence are targeted for developing genotyping methods, the DNA probe designed for one HPV type may cross-react with other non-target types despite the presence of four base mismatches in each pair [25]. Cross-reaction is a major challenge in distinguishing some of the individual genotypes of HPV based on the hybridization technology [26]. As we have demonstrated in this report, certain variants of HPV-16 and HPV-31 share a sequence homology of at least 34 bases downstream of the GP5+ site (Fig. 2). All variants of HPV-6 and HPV-11 share a sequence homology of 34 bases upstream of this region (Fig. 3). Increasing the specificity of the currently available HPV test has been suggested as a potential approach to reduce the number of excessive colposcopic procedures in the USA [27].

Using a modified automated Sanger DNA sequencing for HPV genotyping after nested PCR amplification, we found a 9.9% HPV positive rate in the specimens collected from 2 private gynecology offices primarily serving the local residents with a population 50,000+ in the rural and suburban United States. The true HPV positive rate in this general women population is probably lower because all patients under 30 who had a much higher HPV infection rate were pre-selected with an abnormal Pap cytology before being accepted for HPV testing. In this population, HPV-16 is the most prevalent single genotype found, constituting 19.0% of the HPV isolates, followed by HPV-52, -18 and -59 in decreasing order (Additional file 1, Table 1).

Of the 2,020 cervicovaginal specimens, 5.9% (119 cases) contained at least 1 "high-risk" HPV genotype while only 0.2% (4 cases) showed an HSIL in cytology (Additional file 1, Table 1). Of the 4 HSIL cases, 2 were associated with an HPV-16, 1 with an HPV-39 and 1 with an HPV-69. HPV-69 is not included in the Digene HC2 "high risk" group, but has been shown to be of high risk in HIV-infected patients [28]. No invasive cancer was detected.

If HSIL cytology were used as the endpoint for clinical evaluation, the clinical specificity for HSIL of one-occasion "high-risk" HPV positivity by the DNA sequencing method would be (2020-119)/(2020-4) = 94%; the clinical sensitivity would be 4/4 = 100%; the negative predictive value (2020-119)/(2020-119 + 0) = 100%; and the positive predictive value (*ppv*) would be 4/(4+115) = 3.4%. This extremely low *ppv *of an HPV DNA sequencing assay, even with a high 94% clinical specificity, emphasizes the reality that using one-occasion HPV testing to formulate guidelines for management triage is bound to generate excessive colposcopic procedures [27] in populations with a low cervical cancer prevalence.

In this series, even the most "high-risk" HPV-16 infection is rarely associated with cellular dysplasia. Of the 38 specimens positive for HPV-16 DNA, 2 cases (5.3%) show a HSIL as mentioned above, 2 a LSIL and 5 an ASCUS cytology. The remaining 29 HPV-16 isolates (76.3%) were associated with a reactive or a normal Pap cytology (Additional file 1, Table 1). It seems to be more appropriate to use this virology test for referring patients with an ambiguous Pap cytology result and persistent high-risk HPV infection to colposcopy [29].

Since the introduction of the liquid-based Pap cytology testing into clinical practice, there has been an increasing demand for performing Pap cytology, HPV detection, HPV genotyping, and the detection of *C trachomatis *and *N gonorrhoeae *with one specimen collected in a single container.

In clinical practice, most of the cervicovaginal samples from patients presenting with symptomatic or acute gonococcal infections are collected by swabs which are usually submitted for bacteriological culture and Gram staining. NAA tests for *N gonorrhoeae *in liquid-based Pap cytology specimens are usually requested when the patients present with subclinical or asymptomatic gonococcal infections. Because the traditional cell culture method for *C trachomatis *detection is no longer readily available, requests for *C trachomatis *nucleic acid tests in the liquid-based specimens are often for both symptomatic and asymptomatic patients. As a result, we have seen mostly "low-positive" results for *N gonorrhoeae opa *gene DNA in the liquid-based Pap cytology specimens with a positive rate of 2.0%, but both "low-positive" and "high positive" cases for *C trachomatis *cryptic plasmid DNA in the same period with a positive rate of 1.4%. There seems to be more asymptomatic *N gonorrhoeae *than asymptomatic *C trachomatis *infections in this community. However, since the present series is small and the patients studied are highly selective, these numbers do no represent infection rates in women living in this population.

Accurate nucleic acid detection of *C trachomatis *and *N gonorrhoeae *in clinical specimens is challenging. Most commercial NAA tests target a DNA segment of the cryptic plasmid DNA for *C trachomatis *detection and the sensitivity of the tests has been high. However, recently a variant of *C trachomatis *with a 377-bp deletion in the cryptic plasmid (GenBank accession no. EF 121757) has emerged, causing probe failures for certain commercial kits which target this deleted segment for their real time PCR assays [30,31]. In our procedure, we choose a species-specific sequence about 3000 bases downstream of the 377-bp deletion site for amplification to avoid the potential failure caused by the 377-bp deletion.

Nucleic acid detection of *N gonorrhoeae *is more complex. All commercial gonococcal NAA tests, including those targeting the *cppB*, *opa *and 16S genes and the porA pseudogene for amplification [32-38], are associated with a less than desirable degree of sensitivity and specificity. The great capacity of *N gonorrhoeae *for genetic variation and recombination is the major cause of this technical complexity. Members of the Neisseria species are capable of taking up exogenous DNA throughout their entire life cycle, causing gonococci acquiring commensal Neisseria DNA sequences, and vice versa [39,40]. Genetic recombination between gonococci can take place in vitro and in vivo [41], turning any given DNA segment into a potential moving target for detection. The *cppB*-based PCR assays are known to have generated false-negative results in populations infected by gonococci lacking the *cppB *gene (non-PAU^- ^subtype) [42,43] For our procedure, we have chosen a DNA strand of 40 bases in the promoter region of the gonococcal *opa *genes for target amplification and as the signature sequence. This short DNA sequence appears to be highly conserved and constant for distinguishing itself from those of other non-gonococcal neisserial *opa *genes, including those of *Neisseria meningitidis *[16].

## Conclusion

DNA sequencing can be used for accurate HPV genotyping and accurate molecular identification of *C trachomatis *and *N gonorrhoeae *in clinical laboratories with molecular biology experience to increase the specificity of these microbiology tests. However, an HPV test even with a high virology specificity may still have a very low positive predictive value if a precancer lesion is used as the endpoint for evaluation in a population with low cervical cancer prevalence.

## Competing interests

The corresponding author, Dr. S. H. Lee, declares that he is a shareholder and the president of HiFi DNA Tech, LLC, a company that developed the low temperature PCR technology. The other co-authors declare that they have no competing interests.

## Authors' contributions

SHL conceived the study and participated in acquisition, analysis and interpretation of data and in drafting the manuscript. SP participated in direction of the study, reviewing of the abnormal Pap slides, and analysis of the data with its potential clinical application. VSV participated in organization of the study, performing the nested PCR, performing the automated DNA sequencing, analyzing the sequencing data and alignment of the computer-generated DNA sequences with those stored in the GenBank to achieve the final DNA identification.

All authors have read and approved the revised version of the manuscript.

## Pre-publication history

The pre-publication history for this paper can be accessed here:



## Supplementary Material

Additional file 1 **Table 1**. HPV genotypes correlated with Pap Cytology 200 HPV+ among 2,020 patients visiting private gynecologists in Milford, CT Click here for file
