# Supplementary material for: Molecular tests for human papillomavirus (HPV), Chlamydia trachomatis and Neisseria gonorrhoeae in liquid-based cytology specimen
Source: BMC Womens Health. 2009 Apr 9;9:8. doi: 10.1186/1472-6874-9-8 (PMC2672071; doi:10.1186/1472-6874-9-8)
Supplement: Additional file 1 — Table 1. HPV genotypes correlated with Pap Cytology 200 HPV+ among 2,020 patients visiting private gynecologists in Milford, CT [file 1472-6874-9-8-S1.doc]

**Table 1**

HPV genotypes correlated with Pap Cytology

200 HPV+ among 2,020 patients visiting

private gynecologists in Milford, CT

__________________________________________

**HPV**  **Nos.** **PAP CYTOLOGY**_________

**type**_ **Cases** _ HSIL LSIL ASCUS <ASCUS

6 9 1 2 6

11 1 1

16 38 2 2 5 29

18 14 3 8 3

31 7 2 1 4

33 4 2 2

35 4 2 2

39 4 1 1 2

40 2 1 1

45 6 3 3

52 17 1 5 11

53 2 1 1

54 7 1 6

56 8 1 2 5

58 4 1 2 1

59 10 3 1 6

61 3 3

62 4 4

66 5 2 2 1

67 2 2

68 0

69 1 1

70 5 5

71 2 2

72 3 3

73 9 1 2 6

74 1 1

81 6 2 4

83 1 1

84 3 3

91 1 1

M18 2 1 1

M16,18 1 1

M others 14 3 2 9

Total 200 4 29 44 123

HPV+, positive for human papillomavirus DNA.

HPV type, HPV genotypes validated by L1 DNA sequencing, partial.

Underlined = HPV genotypes targeted by Digene HC2 “high-risk” kit.

M18 = mixed infection with HPV 18 identified by type-specific sequencing primer.

M16,18 = infection with HPV 16 and HPV 18 identified by type-specific sequencing primers.

HSIL, high-grade squamous intraepithelial lesion.

LSIL, low-grade squamous intraepithelial lesion.

ASCUS, atypical squamous or glandular cells of undetermined significance; possible LSIL.

<ASCUS, a reactive process is favored ( Bethesda System [24] ) or negative in women age 30 or older.
